# Novel Frataxin Isoforms May Contribute to the Pathological Mechanism of Friedreich Ataxia

**DOI:** 10.1371/journal.pone.0047847

**Published:** 2012-10-17

**Authors:** Haiyan Xia, Yun Cao, Xiaoman Dai, Zvonimir Marelja, Di Zhou, Ran Mo, Sahar Al-Mahdawi, Mark A. Pook, Silke Leimkühler, Tracey A. Rouault, Kuanyu Li

**Affiliations:** 1 Jiangsu Key Laboratory of Molecular Medicine, Medical School of Nanjing University, Nanjing, China; 2 State Key Laboratory of Pharmaceutical Biotechnology, Nanjing University, Nanjing, China; 3 Institute of Biochemistry and Biology, University of Potsdam, Potsdam, Germany; 4 Division of Biosciences, School of Health Sciences and Social Care, Brunel University, Uxbridge, United Kingdom; 5 Molecular Medicine Program, National Institute of Child Health and Human Development, Bethesda, Maryland, United States of America; Alexander Flemming Biomedical Sciences Research Center, Greece

## Abstract

Friedreich ataxia (FRDA) is an inherited neurodegenerative disease caused by frataxin (FXN) deficiency. The nervous system and heart are the most severely affected tissues. However, highly mitochondria-dependent tissues, such as kidney and liver, are not obviously affected, although the abundance of FXN is normally high in these tissues. In this study we have revealed two novel FXN isoforms (II and III), which are specifically expressed in affected cerebellum and heart tissues, respectively, and are functional *in vitro* and *in vivo*. Increasing the abundance of the heart-specific isoform III significantly increased the mitochondrial aconitase activity, while over-expression of the cerebellum-specific isoform II protected against oxidative damage of Fe-S cluster-containing aconitase. Further, we observed that the protein level of isoform III decreased in FRDA patient heart, while the mRNA level of isoform II decreased more in FRDA patient cerebellum compared to total *FXN* mRNA. Our novel findings are highly relevant to understanding the mechanism of tissue-specific pathology in FRDA.

## Introduction

Frataxin (FXN), a highly conserved protein from bacteria to humans, is important in the biogenesis of iron-sulfur clusters (ISC), prosthetic groups allowing essential cellular functions such as oxidative phosphorylation, enzyme catalysis and gene regulation (see reviews [1], [2]). Reduced expression levels of this protein are sufficient to induce Friedreich ataxia (FRDA), a relentless and currently incurable inherited neurodegenerative disease (see review [3]). Recently many studies suggest that Friedreich ataxia is an epigenetic disorder (see review [4]) and HDAC inhibitors correct frataxin deficiency [5], [6]. It is well accepted that FXN acts as an iron-chaperone within the mitochondrial compartment, and a functional extra-mitochondrial pool of frataxin has also been observed in various human cell types [7]–[10]. Not only FXN, but also other ISC proteins represent a heterogeneous group of proteins with different functional features and different subcellular localizations [11]–[13]. Frataxin is involved in the biosynthesis of ISC not only within mitochondria [14]–[17], but also in the extra-mitochondrial compartment [7] by physically interacting with ISCS/ISD11 and ISCU, components of the Fe/S cluster assembly core machinery [7], [15]–[17].

Extra-mitochondrial isoforms of the central components ISCS and ISCU [12], [18] have been identified in human cells. Little is known about the generation of the extra-mitochondrial FXN in human cells. It is postulated that the extra-mitochondrial isoform represents a cytoplasmic redistribution of frataxin after its mitochondrial processing [7], determined by the molecular size of the cytosolic and mitochondrial isoforms in an SDS-PAGE gel. This indirect sizing technique does not identify exact amino-acid-sequence differences of the N-terminus of FXN, but the observed extra-mitochondrial isoforms are functional.

Cells derived from FRDA patients have a partial defect in ISC-containing proteins, with consequent mitochondrial damage [19], [20], lower ATP production, and impaired iron utilization, leading to mitochondrial iron accumulation [3], [21]. The nervous system and heart are the most severely affected tissues. However, tissues with a high mitochondrial demand, such as kidney and liver, are not obviously affected, although the level of FXN in these tissues is high in healthy individuals (Li K, unpublished data and [22]). And blood cells from Friedreich ataxia patients harbor frataxin deficiency without a loss of mitochondrial function [23]. We suspected that the tissue-specific pathology of FRDA might arise from tissue-specific expression of FXN isoforms and that the extra-mitochondrial isoforms might contribute to molecular pathogenesis.

In this study, we identified two novel tissue specific transcript variants, encoding two isoforms of FXN (isoform II and III), which lack the mitochondrial targeting sequence and are therefore different from the canonical transcript (encoding isoform I). The three isoforms preferentially localized in different compartments. Functional assays revealed that isoform I and II protect Fe-S cluster from oxidative damage, and FXN III acts as a mitochondrial Fe-S-biogenesis enhancer. Our findings suggest a direct mechanism by which FXN deficiency could cause tissue-specific pathology of Friedreich ataxia.

## Results

### Identification of the diverse transcripts of human FXN

**Figure 1:**
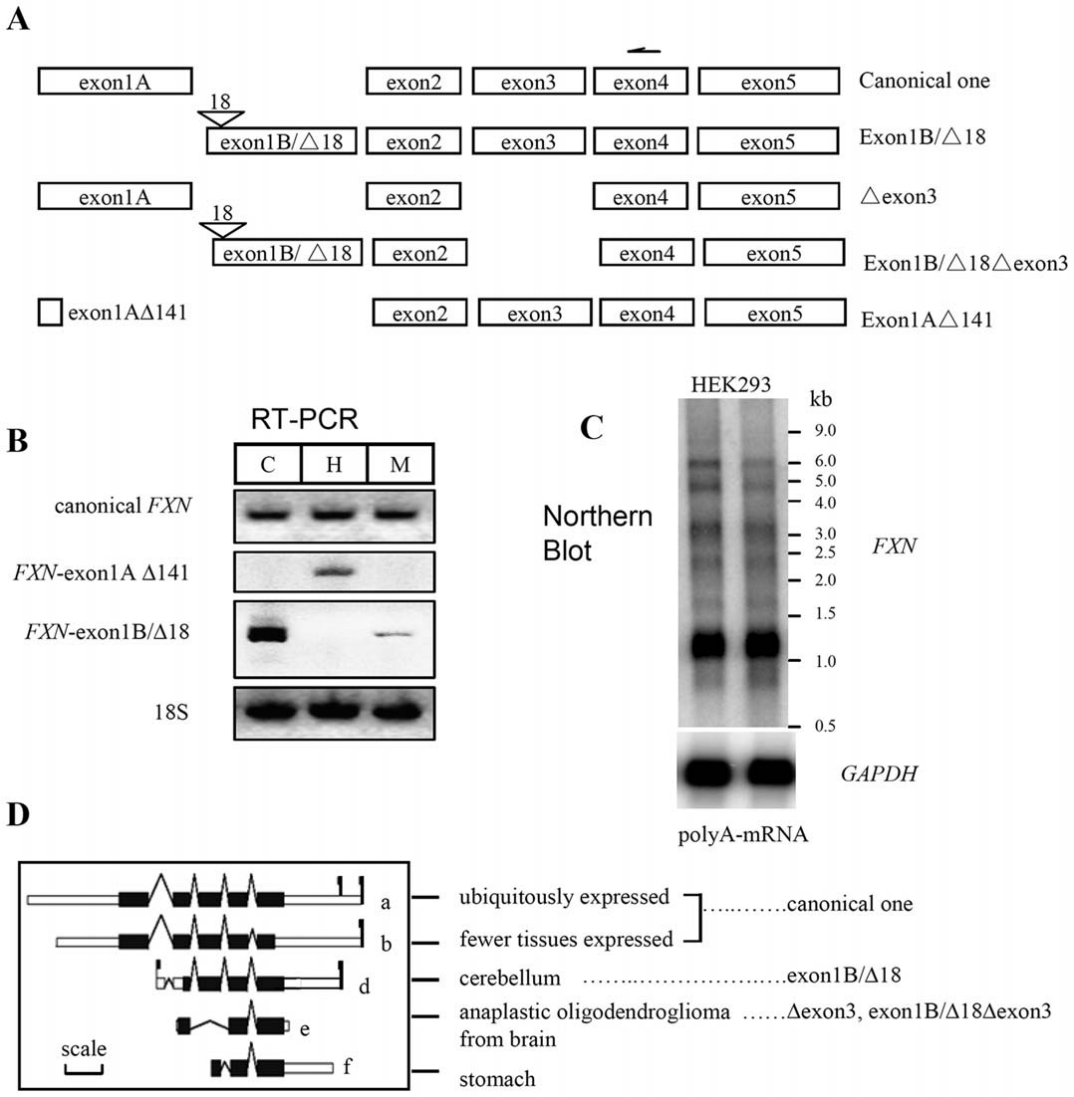
Identification of the diverse transcripts of human *FXN.* (**A**) Schematic diagram of transcripts of human *FXN*. One arrow shows the position of the primer for reverse transcription, locating in exon 4 used for 5′-RACE with RNA from HEK293 cells. PCR products were sequenced to identify the transcripts. Exon1A is the first canonical exon (only the size of coding region shown); exon1A△141, exon1A missing the last 141 nucleotides; exon1B, the novel exon 1 locating in the first intron; exon1B△18, missing 18 nucleotides near the 5′-end of exon1B; △exon3, transcript missing exon3. (**B**) Transcripts of human *FXN* dectected with RT-PCR showed tissue-specific expression pattern. C: cerebellum; H: heart; M: skeletal muscle. (**C**) Diverse transcripts may exist in cells, unveiled by Northern blot. (**D**) Potential *FXN* transcripts from AceView database, verified when human tissues or cell lines were used. Here are only shown transcripts a, b, d, e, and f. See http://www.ncbi.nlm.nih.gov/IEB/Research/Acembly/av.cgi?c=geneid&org=9606&l=2395. Flagged ones (a,b,d) are annotated to be validated. The correlation between the mRNA identified in this study and those annotated in AceView database is linked by spotted lines. Transcript a and b are not distinguishable and are considered as canonical ones because the primer used for 5′-RACE positions within exon 4 of human *FXN*. Scale: 200 bp.

To study whether previously reported FXN isoforms were produced from different transcripts, we performed 5′-RACE with RNA isolated from HEK293 cells, in which a reverse primer (primer 353, sequence see table S1) located in exon 4 was used. The previously observed extra-mitochondrial FXN isoforms could differ in the N-terminus of FXN, so we expected that more than one transcript might be identified. As shown in Figure 1A, we found 7 rational transcripts, which fell into five categories depending on the presence or absence of exon 1A/1B, or exon 3: I) the canonical transcript including exon 1 (now designated as exon1A), 2, 3, 4, and 5; II) transcript without exon 1A, but containing either exon1B or exon1B that lacks 18 nucleotides (exon1BΔ18) from the 5′-end of the transcript; III) canonical transcript that lacks exon 3 (Δexon3); IV) combination of type II and III; V) canonical transcript that lacks 141 nucleotides at the very 3′-end of exon1A. All these transcripts may be generated through alternative splicing or exon skipping except that category II could also be generated through a different transcription start site. To verify the multiple transcripts in case of the artifacts in cell line HEK293, we designed the primers to perform reverse-transcription PCR with commercial total RNA from human tissues including cerebellum, heart, and skeletal muscle. The results showed that all tissues expressed the canonical transcript, whereas heart and cerebellum possessed additional specific bands, which differed in pattern or amount (Figure 1B) from canonical one. We also performed Northern analyses with polyA RNA from HEK293 cells. Multiple *FXN* bands were observed again (Figure 1C), similar to previous results [21], [22].

In order to find more supportive evidence, we searched AceView database. Interestingly, we found a diagram that illustrated comprehensively the alternative transcripts, which are consistent with our findings, but miss exon1AΔ141-containing transcript. The tissues from which the cDNAs were generated are listed and the correlation between the transcripts identified in this study and those annotated in AceView database is linked by spotted lines (Figure 1D).

### Localization of the deduced human FXN isoforms

**Figure 2:**
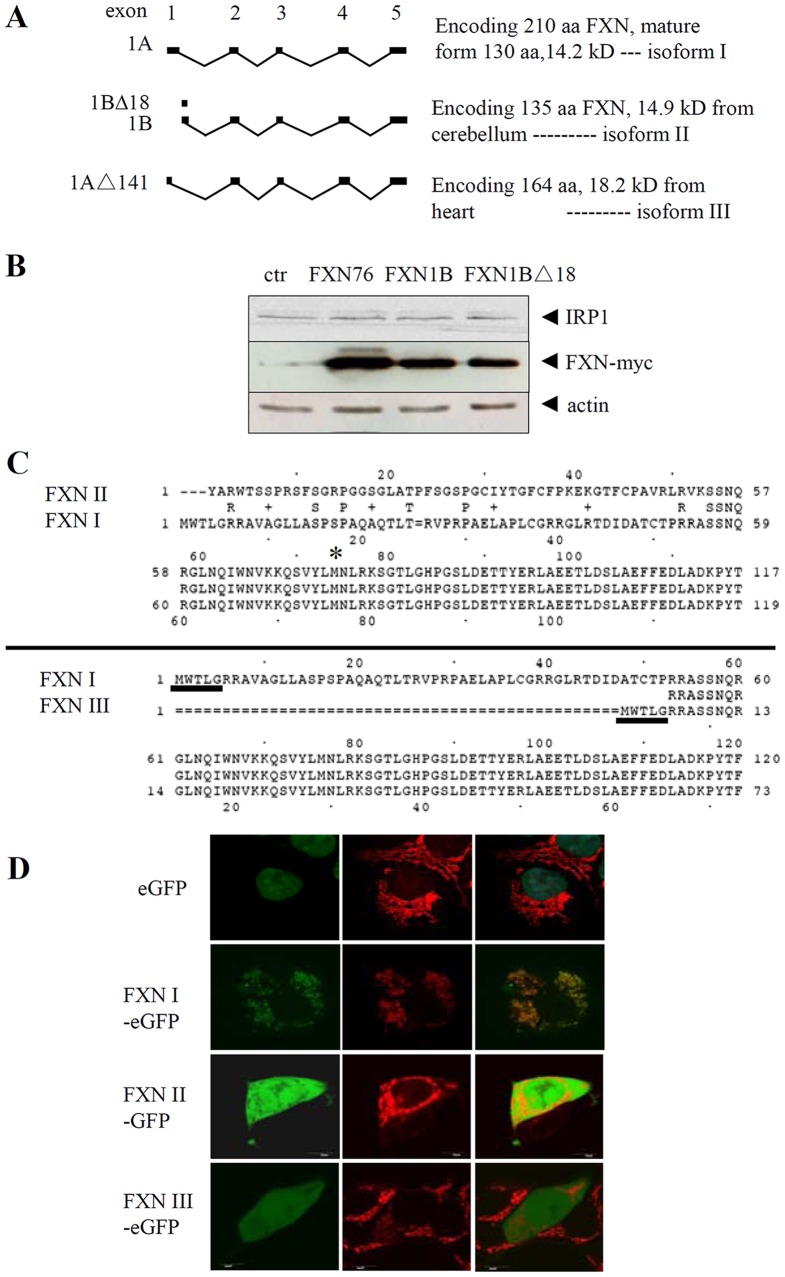
Comparison of putative human FXN isoforms. (**A**) Schematic diagram of the putative FXN isoforms expressed in human heart or cerebellum. (**B**) Western blot showing that exon1B-containing transcript variant encoded a second-AUG initiated isoform FXN76, i.e., FXN II. (**C**) Protein alignment of FXN isoform I, II, III, encoded by transcripts containing canonical exon 1A, exon1B/exon1B△18, and exon1A△141, respectively. Hereafter, exon1B replaces exon1B/exon1B△18. This figure only shows the N-termini of FXN. In the upper panel methionine in the middle of the sequence of FXN is marked with an asterisk, which may be the start codon of the novel FXN transcript containing exon 1B. Underlined amino acid sequences in the bottom panel are identical between the isoform I and III. (**D**) Cellular localization of different isoforms of human FXN. eGFP only: in nucleus; FXN I-eGFP: in mitochondria; FXN II-eGFP: in cytosol and nucleus; FXN III-eGFP: more in nucleus than in cytosol.

To understand the alternative transcripts of human FXN, we first compared the isoforms deduced from the transcript variants. Exon1B/Δ18-containing transcripts could encode a 135-amino-acid isoform (135-aa, FXN isoform II, FXN II) of FXN with an expected molecular size 14.9 kDa, and exon1AΔ141-containing variant is predicted to encode a 164-aa isoform (FXN isoform III, FXN III) of 18.2 kDa. The mature form of canonical isoform (210 aa, FXN isoform I, FXN I), containing N-terminal sequence SGTLG verified by protein sequencing (Columbia University Protein Core Facility), has 130 amino acids (from 81^st^ to 210^th^ aa of the 210-aa precursor) with molecular size 14.2 kDa (Figure 2A). This size of mature form was validated previously by another two groups [9], [24].

Since there is more than one AUG sequence at the 5′-end of exon1B/Δ18-containing transcripts, we cloned three DNA fragments, including exon1B- and exon1BΔ18-containing transcripts and another that encompassed the second AUG (coding for methionine, 76^th^ aa of FXN precursor) of human *FXN* coding region to the stop codon, to see if the first two encode a protein same as the last one as predicted. For simplicity, we designated the three construct-encoded proteins as FXN1B, FXN1BΔ18, and FXN76, respectively. Western results showed that the three constructs encoded the same size of FXN proteins (Figure 2B). Hereafter, FXN II represents exon1B/Δ18-containing transcript-encoded FXN and FXN76. Next, the distinct N-termini of FXN isoforms were aligned (Figure 2C). Full-length FXN I contains a mitochondrial targeting sequence, which is entirely lost in FXN II and partially absent in FXN III. To determine the cellular localization of the isoforms, we constructed plasmids to express FXN-GFP fusion proteins. As expected, FXN I located predominantly in the mitochondria. However, FXN II and III located mainly in the cytosol and nucleus, respectively (Figure 2D). Since the negative control, GFP alone, located mainly in the nucleus, we could not distinguish FXN-isoform localization from the effect of GFP itself although the distinction was clear for FXN I. We then tagged FXN with-myc to detect the localization of FXN isoforms, since the molecular size of myc-tag is much smaller than that of GFP. Again, we observed FXN I in the mitochondria, while FXN II located dominantly in the cytosol and FXN III in the nucleus (Figure S1).

### Tissue-specific expression of human FXN II in the central nervous system (CNS) and FXN III in heart

**Figure 3:**
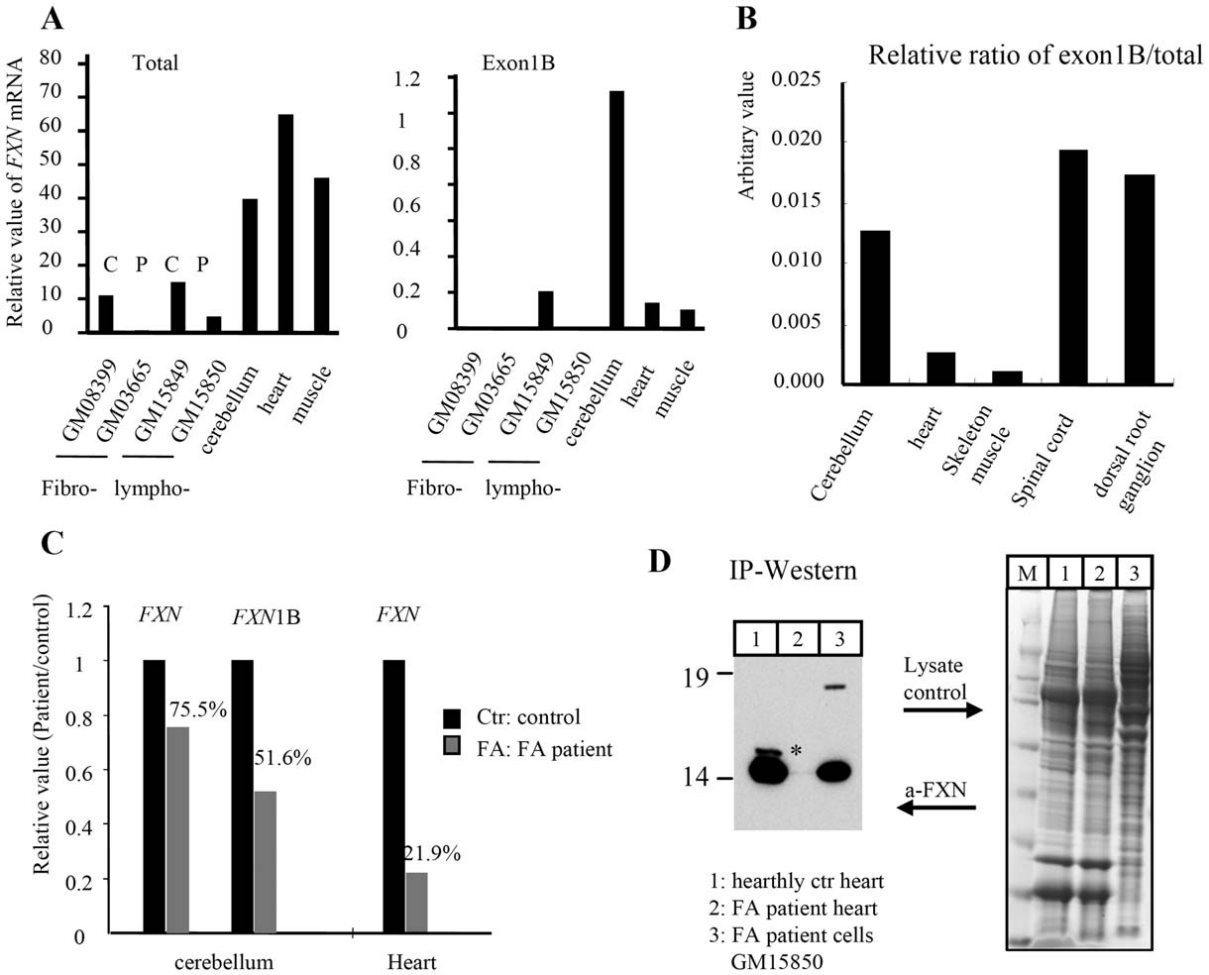
Novel transcripts and/or isoforms of human FXN showed the tissue-specific expression pattern. (**A**) Quantification of frataxin transcripts by qRT-PCR. Total: total mRNA of *FXN*, exon1B: exon1B-containing transcript of *FXN*. Human tissues including cerebellum, heart, and skeletal muscle and cells including fibroblasts (fibro-) and lymphoblasts (lympho-) derived from healthy controls (C) and FRDA patients (P) were analyzed. (**B**) Ratio of transcripts containing exon1B to total *FXN* in human tissues. (**C**) Quantification of *FXN* transcripts in FRDA patient heart and cerebellum, compared to healthy controls. No standard deviation due to 2–3 samples of each tissue from controls or patients is provided. Values are the relative means. (**D**) Immunoprecipitation (IP) of human FXN in a healthy and FA patient heart and patient lymphoblast cells. Lysate before IP was loaded to run a SDS-PAGE gel and then to be stained with Coomassie as a sample control for comparison. Left panel: IP-Western, anti-FXN; right panel: Coomassie-stain sample control. *: a novel heart-bearing FXN band.

To verify the tissue-specific transcripts, we analyzed a variety of tissues, fibroblasts and lymphoblasts derived from healthy controls or FRDA patients by quantitative real-time PCR (qPCR). As shown previously [21], patient cells expressed much lower mRNA levels of *FXN* than control cells, and normal heart expressed the highest levels of *FXN* among the tested tissues (Figure 3A, left panel and [22]). In agreement with the data shown in Figure 1B, cerebellum showed the highest abundance of exon 1B-containing transcripts compared to other tissues such as heart and skeletal muscle (Figure 3A, right panel), although it represented only about 1.25% of total transcripts (Figure 3B). When comparing the most affected CNS tissues including cerebellum, spinal cord and dorsal root ganglion, we found much higher expression levels of exon1B-containing *FXN* transcript in CNS tissues than in other tissues (Figure 3B).

To assess whether the levels of different transcripts changed or not in FRDA patients compared with the healthy controls, we performed qPCR to analyze the mRNA levels of FXN in cerebellum and heart, two of the most affected tissues in FRDA patients. The results showed that the overall mRNA levels of *FXN* decreased, consistent with a previous study [22], while expression of the canonical transcript of *FXN* was more diminished in heart (21.9% of WT levels) than in cerebellum (75.5% of WT levels), and that of the exon1B-containing transcript diminished more (51.6% of WT levels) than the exon1A-containing transcript (75.5% of WT levels) in cerebellum (Figure 3C). The exon1B-containing transcript in heart was below levels of detection. These findings illustrated that expression of the exon 1B-containing transcript was limited to, and significantly reduced in, clinically relevant FRDA tissues, such as the cerebellum. Our attempts to similarly quantify the exon1AΔ141-containing transcript in heart tissue were hampered due to lack of suitable unique sequences.

To detect the existence of protein isoforms encoded by the canonical transcript and the two novel transcripts, we performed immunoprecipitation (IP) and western blots with cell lysates from FRDA patient heart tissue or lymphoblast cells. Figure 3D shows that there was one novel band (* marked) in the control heart lysate in addition to the mature mitochondrial form (Figure 3D). The size of this novel band is slightly larger than the mature form, but smaller than the expected size of FXN III. It might be the degraded FXN III because of the instability of FXN III (see below and discussion). Unfortunately, insufficient human cerebellum tissue was available for IP to identify the endogenous CNS isoforms.

### Functional isoforms of human FXN *in vitro*

**Figure 4:**
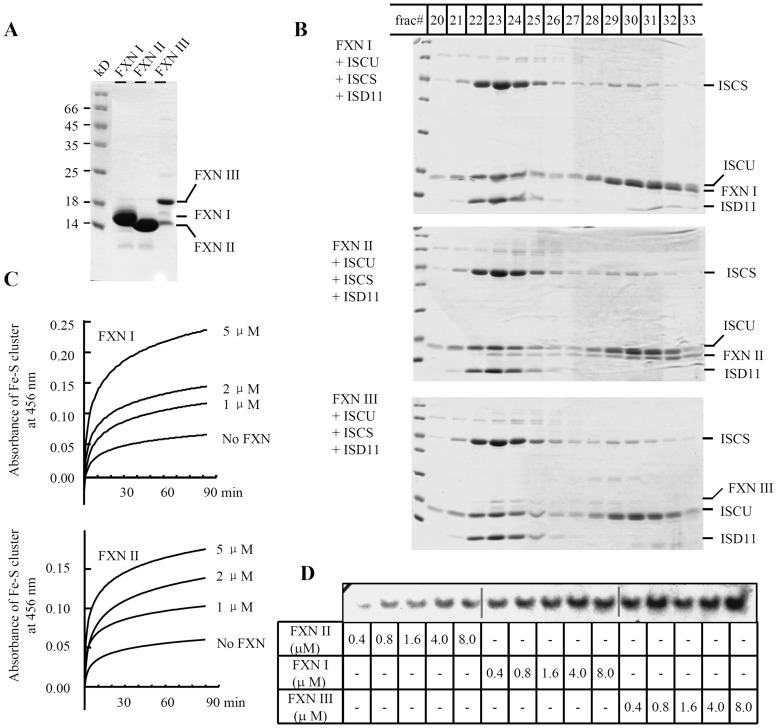
Functional human FXN isoforms facilitating Fe-S cluster assembly *in vitro*. (**A**) FXN isoforms were expressed in *E. coli* and purified to run a SDS-PAGE gel, followed by Coomassie staining. (**B**) Each of the three FXN isoforms could form a complex with other three essential components (ISCS/ISD11, ISCU) of Fe-S cluster assembly *in vitro*. Purified proteins FXN, ISCU, and ISCS/ISD11 were mixed and incubated for 1 hour anaerobically, and then subjected to size exclusion column. The collected fractions were run in SDS-PAGE gels as shown here. (**C**) Fe-S cluster assembly profile at 456 nm within 90 min. (**D**) In-gel aconitase assay of IRP1 after incubation of apo-IRP1 with assembled Fe-S cluster by the complex of four components including FXN, ISCS/ISD11, and ISCU.

To directly assess whether the new isoforms were functional, we used enzymatic assays based on the fact that IRP1 becomes a functional aconitase upon acquisition of a [4Fe-4S] cluster. Three FXN isoforms were overexpressed in *E. coli* and purified as shown in Figure 4A. FXN I and II with expected sizes 14.2 and 14.9 kDa, respectively, ran unusually in a SDS-PAGE gel. FXN I, supposed to run faster than FXN II, actually ran slightly slower, which phenomenon was observed previously [24]. FXN III with expected size 18.0 kDa showed two additional smaller degraded bands, identified by mass spectrometry. Its instability will be discussed in Discussion. To test whether FXN isoforms were able to form a core complex with ISCU, ISCS/ISD11, we also purified the components including human mitochondrial mature forms of ISCU and ISCS/ISD11. We found that these four core components were able to form a complex as shown in Figure 4B, consistent with recently reported results [14]–[16]. Two-component (ISCU+ISCS) or three-component (ISCU+ISCS/ISD11) complexes were easily detected (Figure S2 and Figure 4B, best visualized in later fractions of the middle panel), similar to complexes of these proteins previously identified *in vitro* and *in vivo* in mitochondria and cytosol [11], [12]. Then we asked whether this complex was relevant to facilitate Fe-S cluster assembly. Fe-S cluster was monitored during the assembly and an in-gel aconitase assay was performed to detect the assembled transferred Fe-S. Surprisingly, recording of Fe-S cluster assembly in the presence of FXN III failed due to the obvious precipitation in the reaction, so Figure 4C only shows the kinetics in the presence of FXN I and II. Isoform I showed higher activity than FXN II although their efficiency was comparable. In-gel aconitase assays revealed that three FXN isoforms facilitated Fe-S cluster assembly with an efficiency order of FXN III as the highest, then FXN I in the middle, and then FXN II as the least (Figure 4D), which was consistent with spectrum scan data after iron sulfur cluster assembly with relative low concentration of FXN isoforms (2 µM, Figure S3A).

### Functional evaluation of human FXN isoforms in cell lines

**Figure 5:**
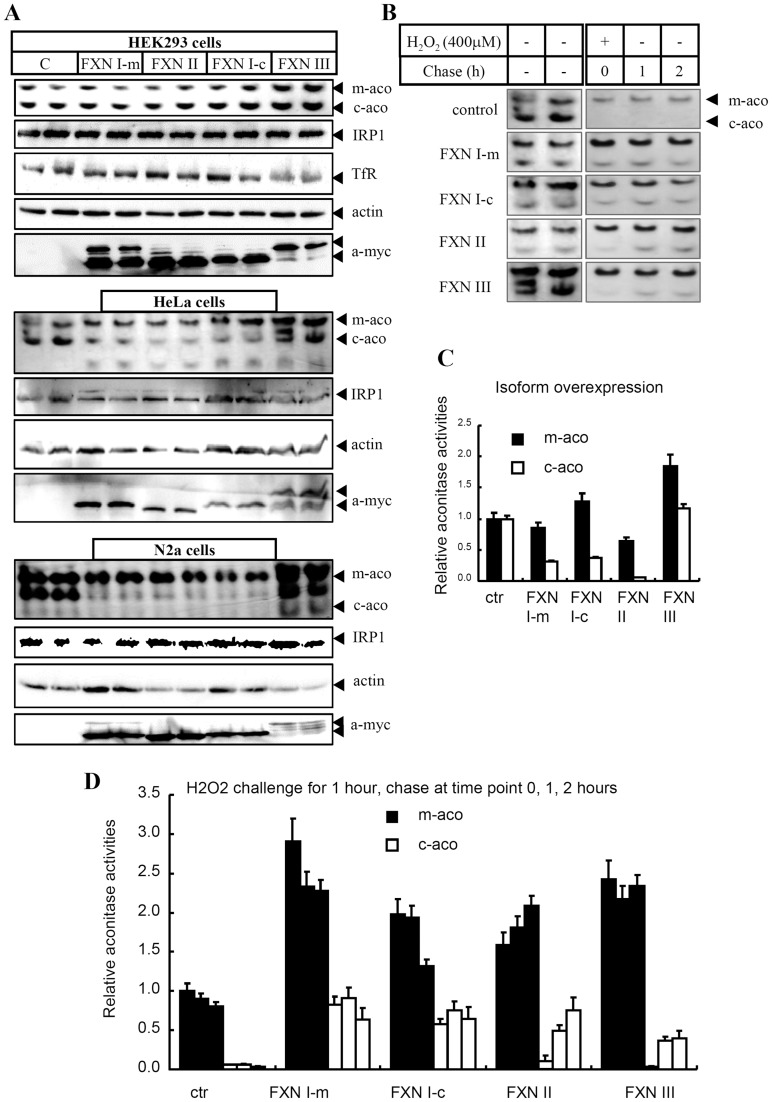
Functional assays of human FXN isoforms in cell lines. (**A**) Each of FXN isoforms was over-expressed in cell lines HEK293, HeLa, and N2a, respectively. Harvested cells were lysed 48 hours post-transfection for aconitase assay and western blot (see Methods and Materials). For each cell line, the top two panels are aconitase assays; the rest are Western blot results. FXN I-m, full-length mitochondria-localized FXN isoform I; FXN I-c, mature form of FXN isoform I, cytosol-localized. m-aco: activities of mitochondrial aconitase; c-aco: activities of cytosolic aconitase, which is Fe-S containing iron regulatory protein 1(IRP1); TfR: transferrin receptor 1. (**B**) Hydrogen peroxide (400 µM H_2_O_2_) challenge on HeLa cells, in which FXN isoforms were expressed as indicated. Aconitase activities were chased within 2 hours. (**C**) Quantification data of left panel of Figure B. (**D**) Quantification data of right panel of Figure B. Both (**C**) and (**D**) are expressed as mean ± SD from three independent experiments.

To further characterize the human FXN isoforms, we expressed the isoforms in different cell lines including HEK293, HeLa, and N2a. Any effect of FXN isoforms on Fe-S cluster biogenesis, or on iron metabolism, could be monitored by the in-gel assay because both cytosolic (c-aco) and mitochondrial (m-aco) aconitases function as sensors of Fe-S cluster biogenesis in both compartments of mammalian cells. Therefore, this assay was performed to evaluate the function of FXN isoforms. Over-expression of full-length (FXN I-m) and mature (FXN I-c) isoform I and FXN II all showed similar negative effects on aconitase activities, probably due to the general toxicity, in agreement with the observation in which the mature form of FXN without a mitochondrial targeting sequence could fully replace full-length FXN [7], [10], [25]. However, the adverse effect was only rather mild in HEK293 cells, but severe in HeLa and N2a cells. More interestingly, c-aco activities were much more deleteriously affected than m-aco activities regardless of the localization of the overexpressed FXN isoforms (Figure 5A). Such negative effects of over-expressed human and fruit fly FXN have also recently been reported [26]. In contrast, FXN III over-expression increased both m-aco and c-aco activities (greater than 2 folds) in all tested cell lines (Figure 5A, 5C), consistent with the *in vitro* assay (Figure 4D), even though the increased abundance of this isoform was the least compared with that of other two isoforms (Figure 5A, α-myc signal in the last two lanes for N2a cells), suggesting that a slight decrease of heart-specific FXN III may have significant impairment effect on aconitase activities in heart.

Treatment of FRDA patients with antioxidants to counteract oxidative stress has been shown to improve neuronal impairment [27]. To further analyze cerebellum-specific FXN II, we first overexpressed FXN isoforms, then treated the cells with hydrogen peroxide (H_2_O_2_) for 1 hour. Aconitase activities were assayed as shown in Figure 5B. Clearly, all of the human frataxin isoforms largely protected the Fe-S-containing aconitase from oxidative damage, particularly the c-aco activity (Figure 5B, 5D), indicating that FXN prevented disassembly of the Fe-S cluster in c-aco to generate apo-IRP1 after H2O2 treatment. Relatively, m-aco activities were much less affected by the H2O2 treatment, suggesting that m-aco is less accessible than c-aco to the effect of H2O2, in agreement with the previous observation [13]. Interestingly, both full-length and mature forms of FXN I, the former dominantly localized in mitochondria and the latter in cytosol (Figure 2D), comparably protected both m-aco and c-aco activities after H2O2 treatment. The protection effect of isoform II, localized largely in cytosol, from disassembly of Fe-S cluster of c-aco was relatively minor. However, the c-aco activities of IRP1 had returned to baseline levels within 1 hr after removal of H2O2, indicating robust *in-vivo* repair/regeneration of the [4Fe-4S] cluster, which was then transferred to apo-IRP1 to gain the aconitase activity. In contrast, isoform III did not efficiently restore c-aco activity within the tested time. Taken together, our results suggest that FXN isoform II, the cerebellum-specific variant, functions more to protect cells from oxidative damage by efficient repair of Fe-S clusters in the cytosol, whereas FXN isoform III, the heart-specific variant, focuses more on Fe-S cluster biogenesis for m-aco activity, which may be physiologically relevant (see discussion).

## Discussion

In this study we have demonstrated that frataxin has various isoforms in addition to the canonical form. We have further shown that at least two of the novel isoforms are expressed in a tissue-specific manner. Using a number of molecular methods, we demonstrate that these two isoforms are functional *in vitro*, forming a complex with three other core-components of the iron-sulfur cluster assembly machinery, ISCU, ISCS, and ISD11, to facilitate Fe-S cluster assembly. A small increase in the abundance of the heart-specific FXN isoform III in cell lines significantly increased the aconitase activity, particularly that of m-aco. In contrast, over expression of cerebellum-specific FXN isoform II protected against oxidative damage of aconitase, particularly c-aco. More importantly, we observed that the levels of FXN, not only the canonical isoform I, but also the newly found isoform III, significantly decreased in FRDA patient heart, while the levels of the isoform-II transcript were comparatively more reduced in FRDA patient cerebellum than that of total *FXN* mRNA. Our novel findings reveal a possible mechanism for the tissue-specific pathology of Friedreich ataxia.

The *in vitro* heterogeneously expressed FXN III displayed different stability from FXN I and II. FXN III was unstable and easily degraded during purification (Figure 4A), whereas FXN I or II always showed a single band in SDS-PAGE gel. This phenomenon was coincidently observed previously by Cowan and his colleagues [28], [29]. FXN investigated in above mentioned paper corresponds to residues 56–210 of human FXN, while FXN III involved in this study missed 47 amino acids from 9^th^ to 55^th^. Very likely, FXN III shares the similar mechanism of “iron mediated self-cleavage” to be degraded *in vitro*. We confirmed that EDTA addition could prevent FXN III from degradation (data not shown). This might explain that heart-specific FXN III showed a smaller size than expected (Figure 3D).

The occurrence of extra-mitochondrial FXN has been debated since Cocozza and colleagues first reported this finding [7]. Subsequent studies have followed up to investigate the potential functions of extra-mitochondrial FXN [8], [10], [25], [30]. Mechanistically, extra-mitochondrial FXN has only been described by comparing the sizes of FXN proteins in SDS-PAGE gels [7], [10]. Here we demonstrate a mechanism of how cytosolic FXN can be generated. Based on the speculation that the difference might be at the very end of N-terminus due to the distinct location of the isoform, we found transcript variants of human *FXN*, two of which encoded isoform II and III, both lacking the mitochondrial signaling peptide. Accordingly, extra-mitochondrial locations were confirmed for both isoforms. And the abundance of *FXN* transcript generating isoform I and II is reduced in the cerebellum of FRDA patients although the reduction level (75.5% and 51.6% of the control levels, respectively) is higher than that reported previously (10–30%) (see review [31]). We could not well explain this divergence from other literature, whereas the expression levels of control FXN varies largely (threefold) in a group of 50 normal people [32] and the reported levels of patient FXN were averaged, mostly in peripheral blood mononuclear cells [23], [33], [34] or fibroblasts/lymphocytes [21], [35],rarely quantified in the affected tissues [36], [37]. The number of the patients in our study is not enough due to the scarcity of the tissues so that the percentage of 75.5% or 51.6% is not a statistical mean of *FXN* levels of patients. Obviously, the real statistical mean needs to be acquired with a large number of healthy controls and FRDA patients.

Isoform II and III localized with cellular compartmental preference for the cytosol or nucleus, respectively, whereas the canonical isoform I localized to mitochondria. In general, all three isoforms displayed protection against oxidative damage of aconitase, particularly that of c-aco, when challenge with H_2_O_2_. The isoform II protective effect on c-aco could be explained by its cytosolic localization and the isoform I protective effect by its minor cytosolic distribution after over expression. Intriguingly, isoform III, which localizes more in the nucleus than in the cytosol, can significantly enhance mitochondrial aconitase activity. This apparent lack of correlation between localization and functional phenotype of isoform III requires further investigation. However, its functional effect and its tissue-specific expression pattern agree with the pathology of affected heart tissue in FRDA patients. Heart, which has a high energy need in accordance with a high rate of Fe-S biogenesis, is one of the tissues maintaining the highest mitochondrial aconitase activity [38]. This activity is deficient in endomyocardial biopsies of FRDA patients, together with deficiency in the activities of the Fe-S cluster-containing subunits of mitochondrial respiratory complexes I, II and III [39], [40]. On the other hand, heart is very sensitive to overloaded iron [41]. Isoform III might be a good mediator to incorporate iron to diminish the toxicity of the overloaded iron in normal heart cells and to promote Fe-S cluster biogenesis. Our *in vitro* data provided evidence in favor of the higher iron binding activities of isoform III than isoform I and II (Figure S3B), in agreement with its high capacity to enhance Fe-S biogenesis (Figure 4D, 5, and S3A). Therefore, even slightly decreased expression of isoform-III may raise the toxicity of overloaded iron in FRDA patients, consistent with ROS production via Fenton chemistry [42].

In summary, our results suggest a novel mechanism for the tissue-specific pathology of Friedreich ataxia. If the existence of a homolog of one of the human FXN isoforms in mouse tissues could be confirmed, using the above findings, a new ‘Fxn isoform-specific defective’ mouse model may be considered to further study FRDA disease mechanisms and therapy in the future.

## Materials and Methods

### Ethics Statement for Human tissues and RNAs

Human tissues were obtained from autopsies of two FRDA patients (47 year-old Caucasian male with GAA repeats of 750/750 and 36 year-old Caucasian female with GAA repeats of 700/700) and two non-FRDA individuals (80 year-old Caucasian male and 82 year-old Caucasian female), in accordance with UK Human Tissue Authority ethical guidelines. Approval has been obtained from the Brunel University School of Health Sciences and Social Care Research Ethics Committee and the Brunel University Human Tissue Act Compliance Committee. The FRDA tissues were obtained from Ataxia UK and non-FRDA tissues were obtained from NDRI (The National Disease Research Interchange) and these organizations hold the details of written consent.

### Cell culture and transfection

Cell lines HEK293, N2a, HeLa were purchased from ATCC (Manassas, VA). Fibroblasts or lymphoblasts derived from healthy control (GM08399) or non-affected carrier (GM15849) and Friedreich ataxia patients (GM03665, GM15850) were obtained from the Coriell Cell Repository (Camden, NJ). HEK293 cells were grown in alpha-modified MEM medium (Sigma, St. Louis, MO), N2a in DMEM, fibroblasts in MEM and lymphoblasts in RPMI 1640 medium, all supplemented with 10% fetal calf serum and 2 mM glutamine (Invitrogen, Carlsbad, CA). For transfection, Fugene 6/HD (Roche, Indianapolis, IN) or lipofectmin 2000 (Invitrogen) was used according to the supplier's manuals.

### DNA manipulation

Plasmid constructs expressing FXN isoforms were generated as following. The PCR products of *FXN* fragments were cloned into plasmid pcDNA3.1(−) with *Xho*I and *Hind*III sites or into plasmid pET24a(+) with *Nde*I and *Xho*I sites or into plasmid peGFP-N1 with *Xho*I and *Bam*HI sites. Plasmid DNA was isolated with Qiagen miniprep or midiprep kits (Valencia, CA) as needed. The right clones were confirmed by sequencing.

A plasmid coexpressing ISCS, which misses the first 55 amino acids of human mitochondrial ISCS precursor (previously described as NFS1Δ1-55 [30]), and ISD11 was generated as follows. By PCR, the restriction sites *Bgl*II and *Xho*I were introduced for cloning the PCR products of ISCS into pACYCDuet-1 (Novagen). The resulting plasmid was designated as pZM90a. In addition, the restriction sites *Nco*I and *Hin*dIII were introduced into the ISD11 cDNA for cloning into the second multiple cloning site of pZM90a. The resulting plasmid was designated as pZM90b.

All primers used for the constructs are listed in table S1.

### Protein expression, purification, size exclusion chromatography, and Fe-S assembly

Three FXN isoforms and ISCU were expressed in *E. coli* strain BL21(DE3) and purified as described previously with some modification [11]. For FXN III, EDTA (5 mM) was added into the lysis buffer before lysing the *E. coli* cells. The concentration of EDTA was then kept 1 mM during the whole purification procedure. The ISCS and ISD11 were heterologously co-expressed in *E. coli* and purified as described previously [11], [30]. For co-expression of ISCU and ISCS or ISCU and ISCS+ISD11, either *E. coli* BL21(DE3) or *E. coli* CL100(DE3) [43] cells lacking endogenous IscS (Δ*iscS*) were used.

Purified ISCS/ISD11, ISCU, and FXN isoform were incubated in an anaerobic chamber in a volume of 500 µl with a molar ratio of 1∶1∶1 for 1 h at room temperature, then immediately applied onto Superdex200 column equilibrated with buffer containing 50 mM Tris, 200 mM NaCl, and 5 µM PLP (pH 8.0). The 500 µl-elution fractions were analyzed by SDS-PAGE.

Fe-S assembly was carried out as described previously [11].

### Quantitative real-time PCR (qPCR), reverse transcriptional PCR (RT-PCR) and Northern blot analysis

Total RNA or mRNA of human tissues were isolated with Trizol (Invitrogen) or purchased from Ambion (Austin, TX). Reverse transcription was performed with RevertAid™ First Strand cDNA Synthesis Kit according to manufacturer's instruction (Fermentas, ShenZhen city, Guangdong province, China). The comparative C_t_ method with SYBR Green was conducted with the ABI 7000 Real-Time PCR System (Applied Biosystems, Foster City, CA). Endogenous GAPDH was used as an internal control for normalization. When mRNA levels of different human tissues were tested, 18S RNA was used as an internal control. All primers used for RT-PCR or qPCR are listed in Table S1.

Northern hybridization was carried out and the blot was exposed to PhosphoImage screen and detected with Typhoon 9200 (GE Healthcare). Full-length *FXN* fragment covering the coding region was labeled with [^32^P]-ATP (Perkin Elmer, Waltham, MA) as a probe. Labeled GAPDH was used as an internal control.

### 5′ Rapid Analysis of cDNA Ends Derived from Full Length RNA (RACE)

5′-RACE was performed using the GeneRacerTM kit (Invitrogen) according to the manufacturer's instructions. Reverse transcription with random hexamers was used to synthesize cDNA. To obtain 5′ ends, the first strand cDNA was amplified using a *FXN* specific primer 353 (see table S1) and the GeneRacerTM 5′ primer. The RACE PCR product was visualized on a 1.5% agarose gel, purified and cloned into the pCR 4-TOPO vector using the TOPO TA cloning kit (Invitrogen). Totally 40 clones were picked for plasmid isolation and further for sequencing.

### Immunoprecipitation and western blotting Analysis

Immunoprecipitation was performed as described previously [21]. In brief, cell lysate was cleaned by incubation with magnetic beads coupled with horseradish peroxidase linked anti-mouse IgG (Invitrogen), then subjected to incubation with FXN antibody (MitoSciences, Eugene, Oregon) coupled magnetic beads. After 2 hours incubation, the beads were washed. The proteins were eluted from the beads for Western.

Proteins were resolved in 12% NUPAGE gels (Invitrogen, cat# NP0342box) and transferred onto nitrocellulose membranes (Invitrogen, cat# IB3010-01). Primary antibodies used were rabbit anti-IRP1 [44] and anti-Ferritin (US Biological Inc., Swampscott, MA), mouse anti-tubulin (Abcam, Cambridge, MA), anti-transferrin receptor (Zymed, San Francisco, CA), and anti-frataxin (MitoScience). The mature frataxin form (∼14 kDa) was quantified for comparison when needed. Western blot band intensities were quantified using program ImageJ. Any change of the intensities was compared with the controls, which value was set as 1.

### In-gel aconitase activity assays

In-gel aconitase activity assays were performed as described previously [13]. Related chemicals used were purchased from Sigma (St Louis, MO).

### Determination of protein localization

FXN constructs were generated by ligation of FXN fragment with peGFP-N1 (Clontech) or pcDNA3.1-myc (self made by adding myc-tag DNA fragment into multiple cloning site of pcDNA3.1(−)). C-terminal GFP- or myc-tagged FXN fusion protein was expressed after transfection. Mitotracker was purchased from Sigma for mitochondrial staining. Localization of FXN-GFP or FXN-myc was determined with Confocal (FV 10i-o, Olympus) by either direct visualizing fluorescence (GFP) or immunofluoresence staining (-myc).

### Statistical analysis

All data are representative of three or more independent experiments. Due to the scarcity of the affected tissues, only 2–3 samples for each tissue were used in this study so that direct comparison between patients and control was conducted with mean only without standard deviation (SD). Statistical analyses for function assays of each isoform were performed by Student's t test for paired samples (see http://danielsoper.com/statcalc3/default.aspx) and expressed as mean ± SD.

## Supporting Information

Figure S1 **Subcellular localization of human FXN isoforms.** C-terminal myc-tagged FXN isoforms were expressed in HEK293 cells. The localization was determined by Confocal.
(TIF)

Figure S2 **Two-component (ISCU+ISCS, panel 1,3) or three-component(ISCU+ISCS/ISD11, panel 4)complexes were detected in a single NI-NTA affinity purification step.** His-tagged human ISCU was over-expressed and purified with NI-NTA resin. Endogenous IscS of *E. coli* (panel 1, 4) and untagged recombinant human ISCS with (panel 4) or without ISD11 (panel 3) could be co-purified with human ISCU, confirmed by mass spectrometry. BL21(DE3): *E. coli* strain for over-expression of recombinant protein; CL100(DE3): *E. coli* strain with *iscS* deletion (Δ*isc*S).
(TIF)

Figure S3 **High iron binding activity of FXN III is correlated with its high capacity for iron-sulfur cluster assembly.** A FXN III is the most efficient for iron sulfur cluster assembly. Anaerobic iron-sulfur cluster assembly in vitro was performed with or without FXN isoforms and the kinetic spectrum was scanned with 15 min interval within 90 min. B Iron content was determined by inductively coupled plasma atomic emission spectroscopy. *E. coli* were cultured in LB growth medium with or without iron addition for FXN over-expression. FXN proteins were purified and subject to iron content determination.
(TIF)

Table S1 **Primer pairs used in this study.**
(DOC)
