# Supplementary figures and images for: Novel Frataxin Isoforms May Contribute to the Pathological Mechanism of Friedreich Ataxia
Source: PLoS One. 2012 Oct 17;7(10):e47847. doi: 10.1371/journal.pone.0047847 (PMC3474739; doi:10.1371/journal.pone.0047847)

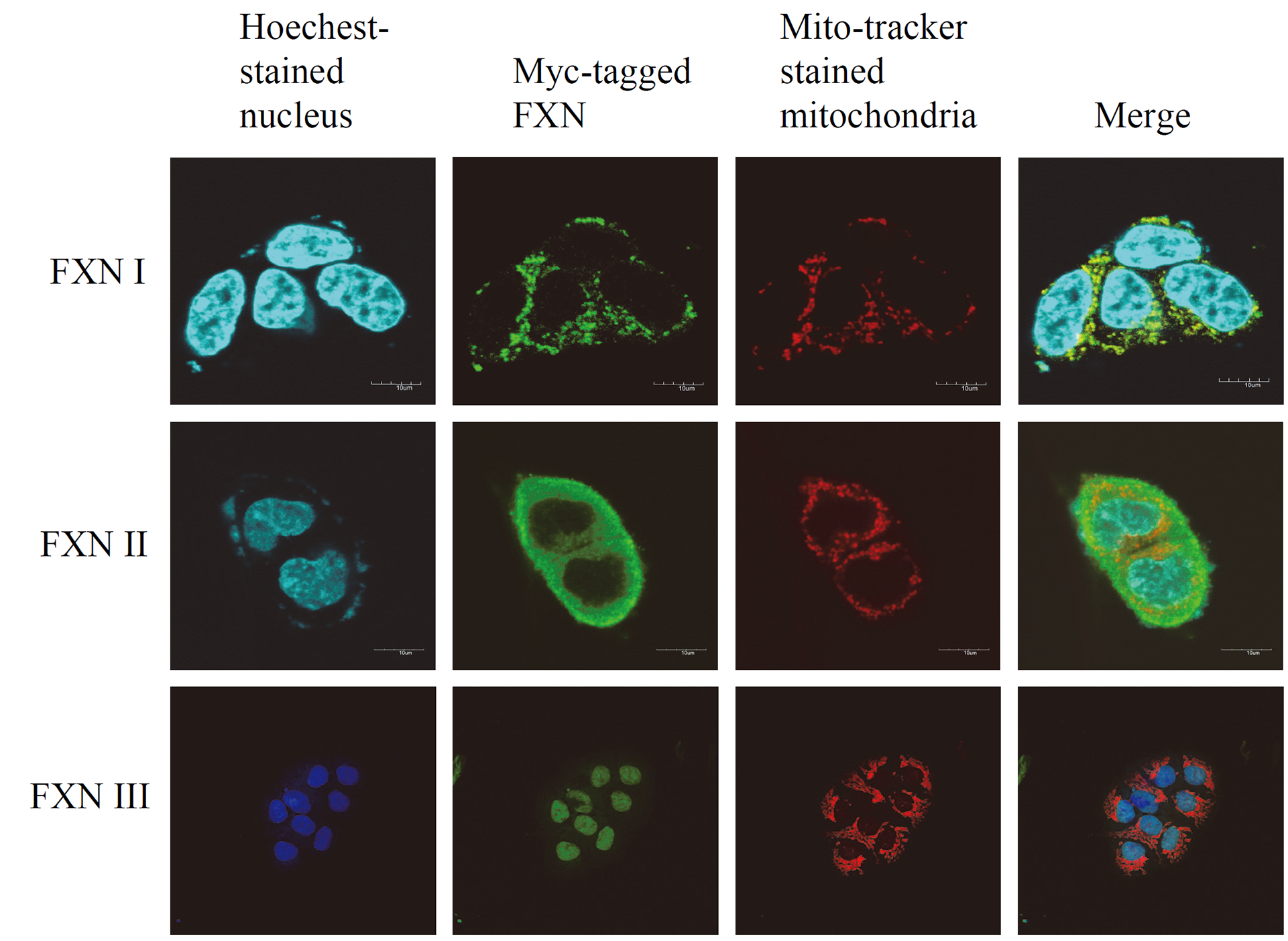

Supplement: Figure S1 — Subcellular localization of human FXN isoforms. C-terminal myc-tagged FXN isoforms were expressed in HEK293 cells. The localization was determined by Confocal. (TIF) [file pone.0047847.s001.tif]

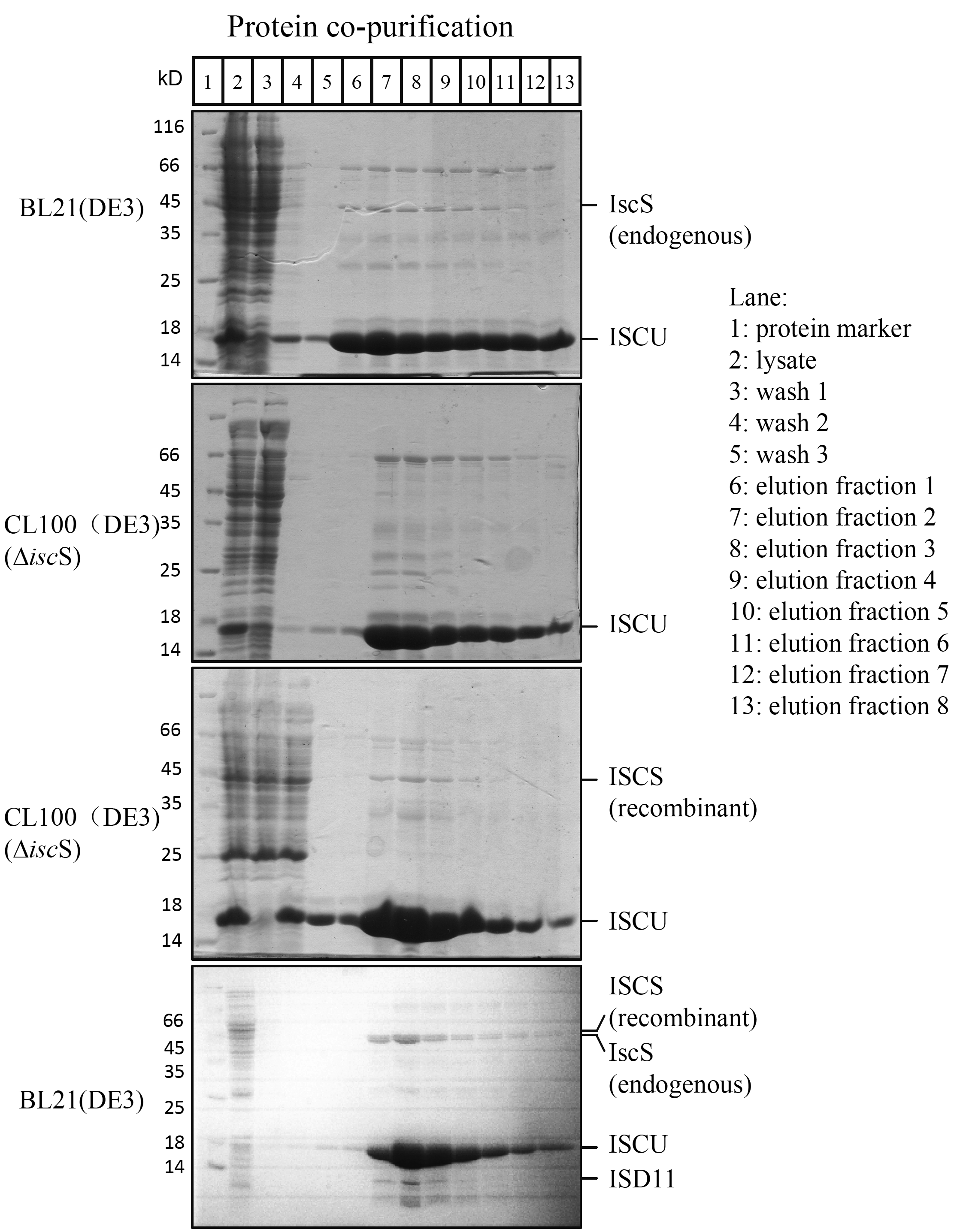

Supplement: Figure S2 — Two-component (ISCU+ISCS, panel 1,3) or three-component(ISCU+ISCS/ISD11, panel 4)complexes were detected in a single NI-NTA affinity purification step. His-tagged human ISCU was over-expressed and purified with NI-NTA resin. Endogenous IscS of E. coli (panel 1, 4) and untagged recombinant human ISCS with (panel 4) or without ISD11 (panel 3) could be co-purified with human ISCU, confirmed by mass spectrometry. BL21(DE3): E. coli strain for over-expression of recombinant protein; CL100(DE3): E. coli strain with iscS deletion (ΔiscS). (TIF) [file pone.0047847.s002.tif]

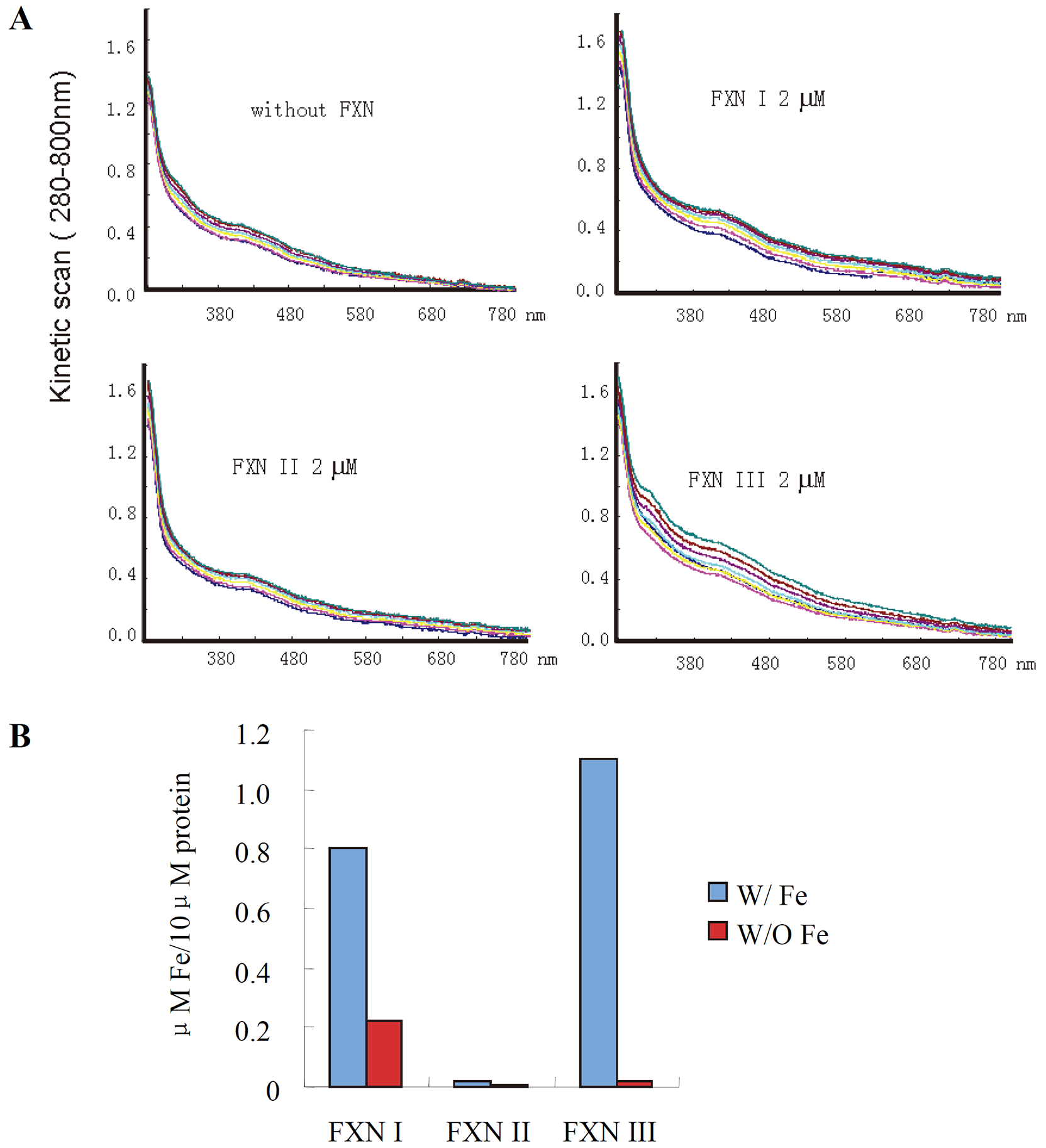

Supplement: Figure S3 — High iron binding activity of FXN III is correlated with its high capacity for iron-sulfur cluster assembly. A FXN III is the most efficient for iron sulfur cluster assembly. Anaerobic iron-sulfur cluster assembly in vitro was performed with or without FXN isoforms and the kinetic spectrum was scanned with 15 min interval within 90 min. B Iron content was determined by inductively coupled plasma atomic emission spectroscopy. E. coli were cultured in LB growth medium with or without iron addition for FXN over-expression. FXN proteins were purified and subject to iron content determination. (TIF) [file pone.0047847.s003.tif]
