# Supplementary material for: Novel Frataxin Isoforms May Contribute to the Pathological Mechanism of Friedreich Ataxia
Source: PLoS One. 2012 Oct 17;7(10):e47847. doi: 10.1371/journal.pone.0047847 (PMC3474739; doi:10.1371/journal.pone.0047847)
Supplement: Table S1 — Primer pairs used in this study. (DOC) [file pone.0047847.s004.doc]

Table S1 Primers used in this study

| Primer name | Sequence (5’-3’) | pair | Purpose |
| --- | --- | --- | --- |
| Primer 375 | AATTAGCTAGCATGGACTCTCGGGC | Primer 375 and 378 | Expression of human full-length FXN (isoform I) or isoform III in mammalian cells |
| Primer 378 | GGGAAGCTTAGCATCTTTTCCGGAATAGG |  |  |
| Primer 376 | GCGGGGCTAGCATGAATTTGAGGAAATCTGG | Primer 376 and 378 | Expression of human FXN isoform II (76^th^ to 210^th^) in mammalian cells |
| Primer 377 | TCTGCTAGCATGTCTGGAACTTTGGGCCACC | Primer 377 and 378 | Expression of human mature FXN (isoform I, 81^st^ to 210^th^) in mammalian cells |
| Primer 368 | TCTCATATGTCTGGAACTTTGGGCCACC | Primer 368 and 370 | Expression of human mature FXN (81^st^-210^th^) in *E. coli* |
| Primer 370 | GACTCGAGAGCATCTTTTCCGGAATAGG |  |  |
| Primer 367 | GCGGGCATATGAATTTGAGGAAATCTGG | Primer 367 and 370 | Expression of human FXN isoform II (76^th^ to 210^th^) in *E. coli* |
| Primer 369 | AATTTCATATGTGGACTCTCGGGCGC | Primer 369 and 370 | Expression of human FXN isoform III in *E. coli* |
| Primer 110 | AAAATCTCGAGCCACCATGGGGACTCTCGGGCGCCGC | Primer 110 and 111 | Determination of localization of FXN isoform I/III |
| Primer 111 | ATCGGATCCCGAGCATCTTTTCCGGAATAGGCC |  |  |
| Primer 182 | TTTTCTCGAGTACGCGCGCTGGACTAGCTCA | Primer 182  and 111 | Determination of localization of FXN isoform II |
| Primer 352 | CCTTGCAGACAAGCCATACA | Primer 352 and 353 | RT-PCR or qPCR of human total *FXN* transcript |
| Primer 353 | CCACTGGATGGAGAAGATAG |  |  |
| FRDA1B-clone10 >A | TACGCGCGCTGGACTAGCTCA | FRDA1B- clone10>A and primer G | RT-PCR or qPCR of human *FXN* transcript variant, encoding isoform II |
| Primer G | GGCTTGTCTGCAAGGTCTTC |  |  |
| Primer I | CGGAGCAGCATGTGGACTCT | Primer I and H | RT-PCR of human *FXN* transcript variant, encoding isoform III |
| Primer H | TGGTTGAGGCCACGTTGGTTC |  |  |
| Primer 296 | TGCACCACCAACTGCTTAGC | Primer 296 and 297 | qPCR of human *GAPDH* |
| Primer 297 | GGCATGGACTGTGGTCATGAG |  |  |
| ZM32_F | GCAGATCTGCGACCTCTCTATATGG | ZM32_F and ZM19_R | Expression of human mitochondrial ISCS (Δ1-55) in *E. coli* |
| ZM19_R | GCCTCGAGCTAGTGTTGGGTCC |  |  |
| LYRM4_5 | GGCCATGGCAGCCTCCAGTCGC | LYRM4_5 and LYRM4_3 | Co-expression of human ISD11 with ISCS in *E. coli* |
| LYRM4_3 | GGAAGCTTCTAGGTCCTGGGCATGTA |  |  |
